# Supplementary material for: Does Very Poor Performance Status Systematically Preclude Single Agent Anti-PD-1 Immunotherapy? A Multicenter Study of 35 Consecutive Patients
Source: Cancers (Basel). 2021 Mar 2;13(5):1040. doi: 10.3390/cancers13051040 (PMC7958129; doi:10.3390/cancers13051040)
Supplement: Supplementary file 1 [file cancers-13-01040-s001.pdf]

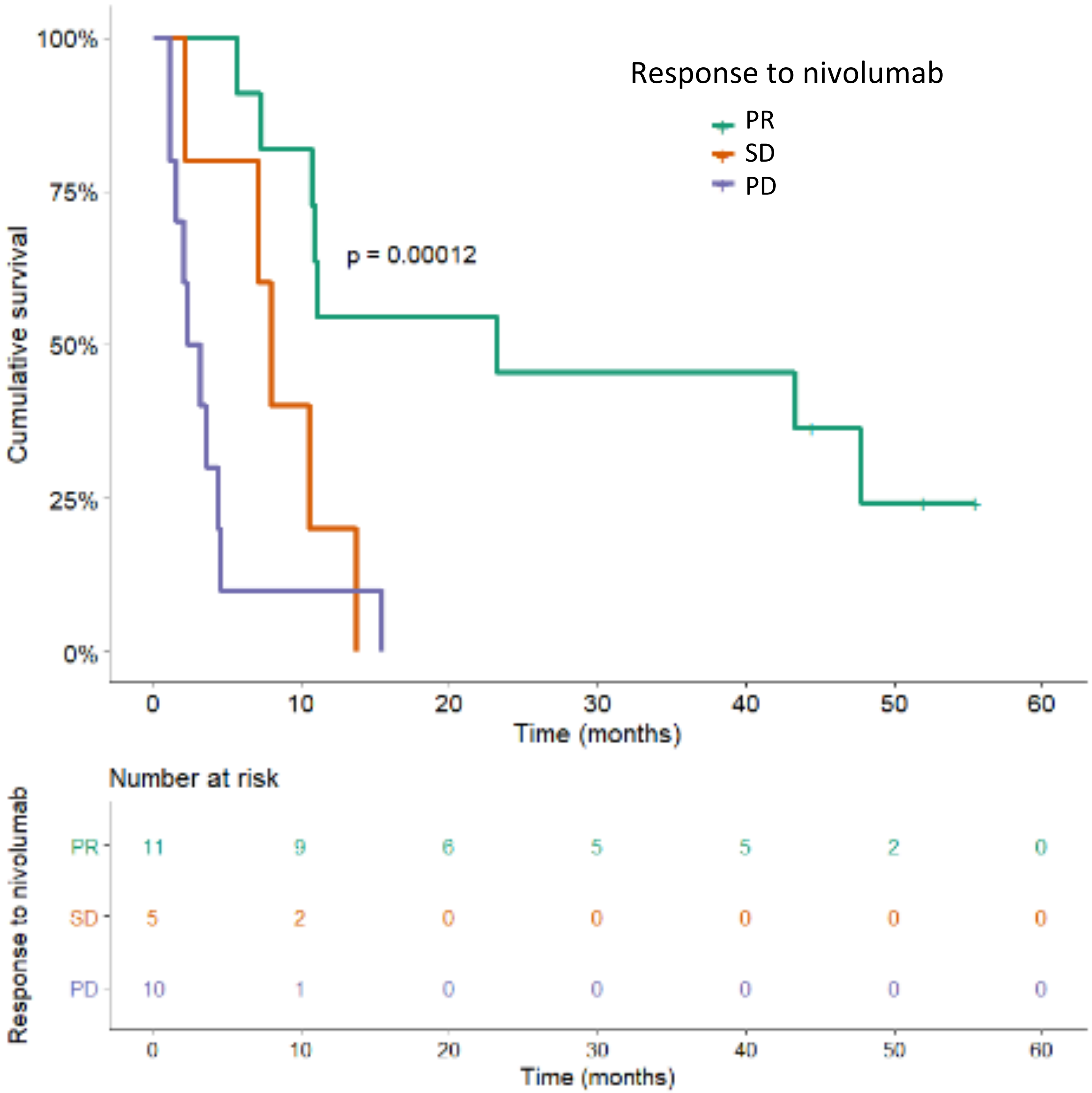

Response to nivolumab
PR
SD
PD
p = 0.00012
100%
75%
50%
25%
0%
Cumulative survival
0
10
20
30
40
50
60
Time (months)
Number at risk
Response to nivolumab
PR 11 9 6 5 5 2 0
SD 5 2 0 0 0 0 0
PD 10 1 0 0 0 0 0
Time (months)

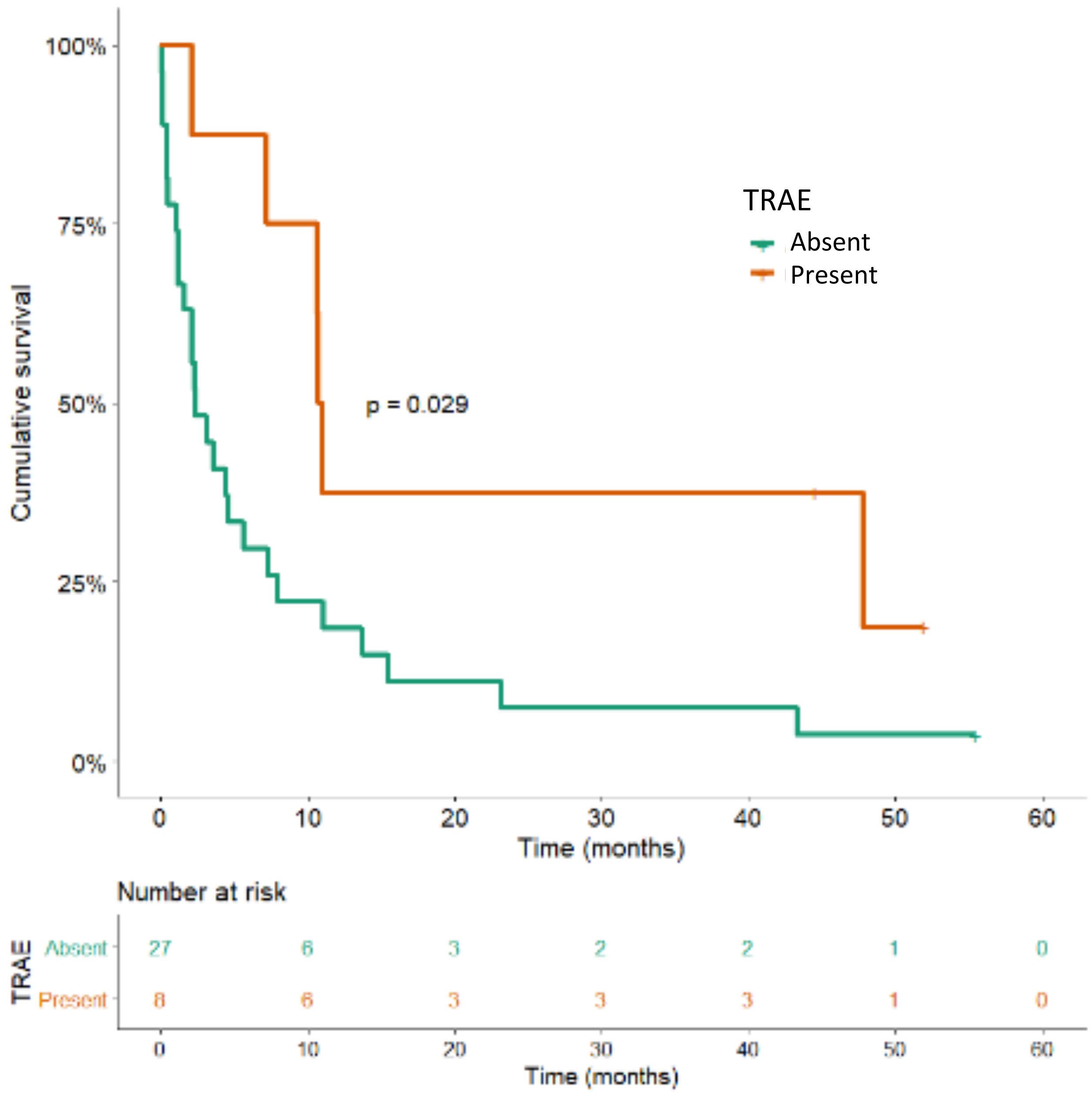

100%
75%
50%
25%
0%
Cumulative survival
TRAE
Absent
Present
p = 0.029
0
10
20
30
40
50
60
Time (months)
Number at risk
TRAE
Absent 27 6 3 2 2 1 0
Present 8 6 3 3 3 1 0
0
10
20
30
40
50
60
Time (months)

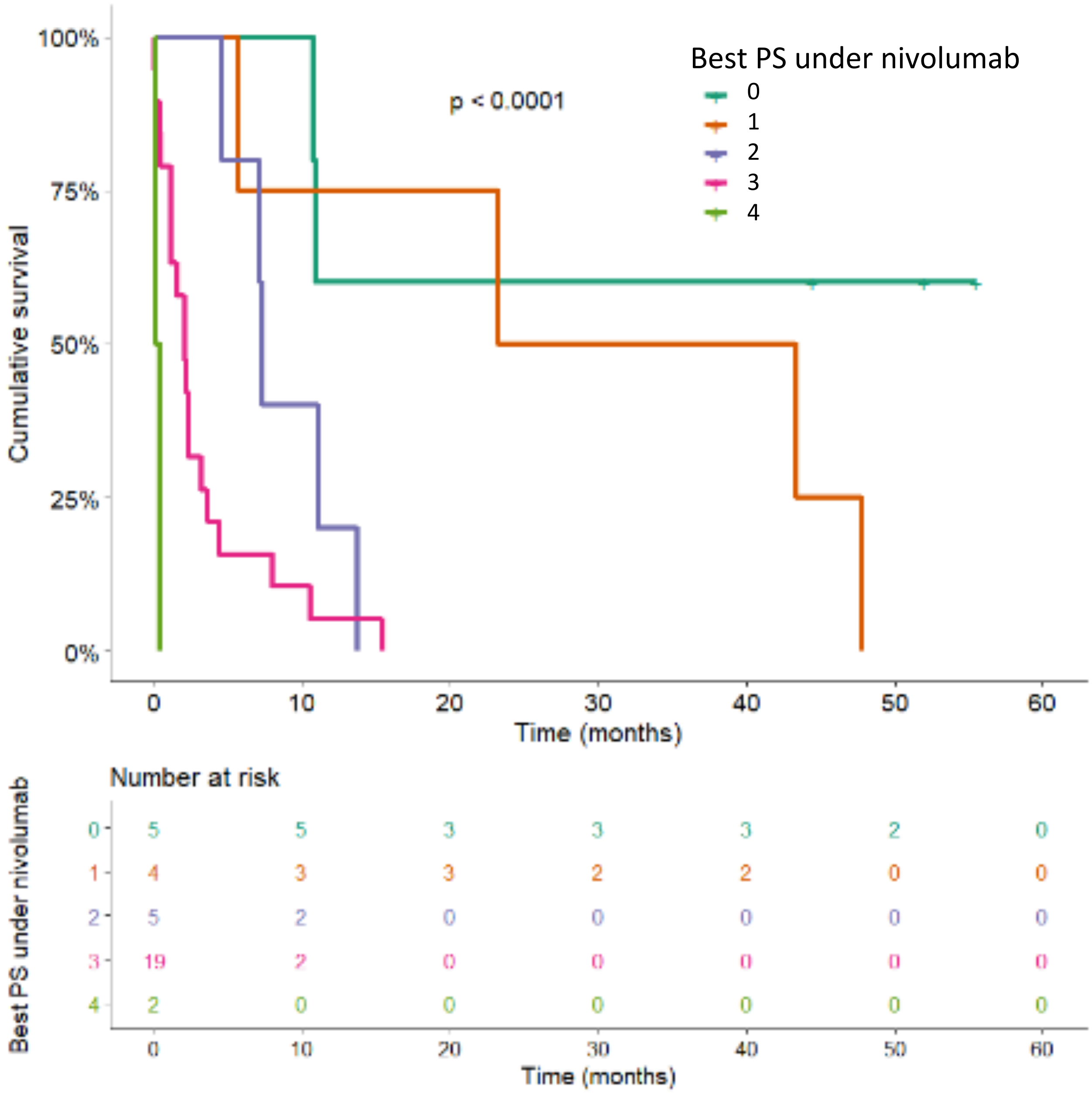

Best PS under nivolumab
0
1
2
3
4
p < 0.0001
100%
75%
50%
25%
0%
Cumulative survival
0
10
20
30
40
50
60
Time (months)
Number at risk
Best PS under nivolumab
0 5 5 3 3 3 2 0
1 4 3 3 2 2 0 0
2 5 2 0 0 0 0 0
3 19 2 0 0 0 0 0
4 2 0 0 0 0 0 0
0
10
20
30
40
50
60
Time (months)

**Supplementary Data Table 1.** Overall Response Rate by PD-L1 Status

| | Patients | | PD-L1 status (TPS) | | | | |
|---|---|---|---|---|---|---|---|
| | N = 34* | | < 1% N = 11 | | ≥ 1% N = 23 | | p |
| | n | % | n | % | N | % | |
| **Objective response** | 11 | 32 | 2 | 18 | 9 | 39 | 0.271 |
| **No objective response†** | 23 | 68 | 9 | 82 | 14 | 61 | |

*In one case, tumor material was insufficient, and the analysis could thus not be performed.

†In the intent-to-treat series, no objective response included stable disease and progressive disease in evaluable tumors and non-evaluable tumors regardless of the cause.

PD-L1, programmed death ligand 1; TPS, tumor proportion score.

| | | | Univariable analysis | | | | Multivariable analysis | | | |
|---|---|---|---|---|---|---|---|---|---|---|
| **Variables** | **N** | **Median OS (mo)** | **95% CI** | **HR** | **95% CI** | **p value (Log Rank)** | **Wald** | **aHR** | **95% CI** | **p value** |
| **** Age (years)*** | 35 | | | | | | | | | |
| < 65 | 16 | 5.7 | 0.4-11.0 | - | | 0.092 | | | | |
| ≥ 65 | 19 | 2.1 | 1.4-2.9 | 1.8 | 0.9-3.7 | | | | | |
| ***Gender*** | 35 | | | | | | | | | |
| Female | 12 | 7.2 | 0-15.9 | - | | 0.729 | | | | |
| Male | 23 | 3.6 | 0.4-6.8 | 1.1 | 0.6-2.4 | | | | | |
| **** Smoking status (PY)*** | 34 | | | | | | | | | |
| ≥ 20 | 27 | 5.7 | 0.8-10.5 | - | | **0.014** | 8.6 | - | - | **0.003** |
| < 20 | 7 | 1.6 | 0-4.6 | 2.9 | 1.2-7.0 | | | 4.8 | 1.7-13.8 | |
| ***Number of metastatic sites*** | 34 | | | | | | | | | |
| < 3 | 22 | 4.4 | 0-13.9 | - | | 0.120 | | | | |
| ≥ 3 | 12 | 3.6 | 0-7.7 | 1.8 | 0.9-3.9 | | | | | |
| **** Liver metastasis*** | 34 | | | | | | | | | |
| No | 25 | 7.3 | 1.7-12.9 | - | | **0.047** | | | | |
| Yes | 9 | 2.1 | 0.5-3.8 | 2.2 | 1.0-5.1 | | | | | |
| **** Brain metastasis*** | 34 | | | | | | | | | |
| No | 24 | 8.0 | 0.4-15.6 | - | | **0.003** | 10.4 | - | - | **0.001** |
| Yes | 10 | 2.1 | 0.6-3.6 | 3.5 | 1.5-8.5 | | | 5.2 | 1.9-14.3 | |
| ***Histological type*** | 35 | | | | | | | | | |
| Adenocarcinoma | 23 | 4.6 | 0.7-8.5 | - | | | | | | |
| Squamous cell carcinoma | 7 | 3.6 | 0.4-6.9 | 0.9 | 0.4-2.1 | 0.947 | | | | |
| Other | 5 | 0.5 | 0.3-0.7 | 1.0 | 0.3-2.8 | | | | | |
| ***PD-L1 status (TPS)*** | 34 | | | | | | | | | |
| ≥ 50 | 14 | 4.6 | 0-9.2 | - | | | | | | |
| 1-49 | 9 | 7.2 | 0-22.1 | 1.3 | 0.5-3.1 | 0.673 | | | | |
| 0 | 11 | 2.3 | 0.7-4.0 | 1.5 | 0.6-3.3 | | | | | |
| ***KRAS mutation*** | 32 | | | | | | | | | |
| Yes | 8 | 7.3 | 0-14.6 | - | | 0.223 | | | | |
| No | 24 | 2.3 | 0.5-4.1 | 1.7 | 0.7-4.0 | | | | | |
| ***First-line therapy*** | 35 | | | | | | | | | |
| Chemotherapy (platinum doublet) | 29 | 4.6 | 0.9-8.2 | - | | 0.230 | | | | |
| ICI (Single anti-PD1, Nivolumab) | 6 | 0.5 | 0-2.8 | 1.7 | 0.7-4.3 | | | | | |
| ***PS before nivolumab initiation*** | 35 | | | | | | | | | |
| 3 | 29 | 4.4 | 1.9-6.9 | - | | 0.941 | | | | |
| 4 | 6 | 1.1 | 0-9.3 | 1.0 | 0.4-2.7 | | | | | |
| ***Best PS under nivolumab*** | 35 | | | | | | | | | |
| 0 | 5 | NR | - | - | | | | | | |
| 1 | 4 | 23.2 | 0-60.2 | 2.7 | 0.5-15.1 | | | | | |
| 2 | 5 | 7.3 | 7.1-7.5 | 8.3 | 1.3-53.4 | **$< 10^{-6}$** | | | | |
| 3 | 19 | 2.1 | 1.3-2.9 | 23.0 | 4.1-128.6 | | | | | |
| 4 | 2 | 0.1 | - | 201.5 | 17.1-2370.2 | | | | | |
| ***Response to nivolumab*** | 35 | | | | | | | | | |
| Partial response | 11 | 23.2 | 0-58.2 | - | | | | | | |
| Stable disease | 5 | 8.0 | 6.2-9.8 | 3.8 | 1.1-13.6 | | | | | |
| Progressive disease | 10 | 2.3 | 0.6-4.0 | 8.7 | 2.8-26.6 | **$< 10^{-7}$** | | | | |
| Not evaluable | 9 | 0.4 | 0.3-0.5 | 60.5 | 13.8-264.6 | | | | | |
| ***Immune-related adverse events*** | 35 | | | | | | | | | |
| No | 27 | 2.3 | 0.6-4.1 | - | | | | | | |
| Yes | 8 | 10.7 | 10.3-11.2 | 0.4 | 0.2-0.9 | **0.029** | | | | |

**Supplementary Table 2.** Detailed univariable (Kaplan-Meier) and multivariable analysis (Cox propotional hazards) results.

* variable introduced in multivariable analysis

OS, overall survival

95% CI, 95% confidence interval

HR, hazard ratio

aHR, adjusted hazard ratio

NR, not reached

PY, pack-years

PD-L1, programmed death ligand 1

TPS, tumor proportion score

KRAS, Kirsten rat sarcoma oncogene

PS, Performance status

NSCLC, non-small cell lung cancer
